# Time to Reconsider Diverse Ways of Working in Japan to Promote Social Distancing Measures against the COVID-19

**DOI:** 10.1007/s11524-020-00464-4

**Published:** 2020-06-30

**Authors:** Shuhei Nomura, Daisuke Yoneoka, Yuta Tanoue, Takayuki Kawashima, Shoi Shi, Akifumi Eguchi, Hiroaki Miyata

**Affiliations:** 1 Department of Health Policy and Management School of Medicine, Keio University, 35 Shinanomachi, Shinjuku-ku, 160-8582 Tokyo Japan; 2 Department of Global Health Policy, Graduate School of Medicine, The University of Tokyo, Tokyo, Japan; 3 Graduate School of Public Health, St. Luke’s International University, Tokyo, Japan; 4 Institute for Business and Finance, Waseda University, Tokyo, Japan; 5 Department of Mathematical and Computing Science, Tokyo Institute of Technology, Tokyo, Japan; 6 Department of Systems Pharmacology, Graduate School of Medicine, The University of Tokyo, Tokyo, Japan; 7 Laboratory for Synthetic Biology, RIKEN Center for Biosystems Dynamics Research, Osaka, Japan; 8 Department of Sustainable Health Science, Center for Preventive Medical Sciences, Chiba University, Chiba, Japan

The World Health Organization declared the outbreak of coronavirus disease 2019 (COVID-19) on March 11, 2020, highlighting the importance of preventive actions to limit the spread of infections [1]. These measures can be generally divided into those that require individual-level efforts (e.g., handwashing, mask-wearing) and those that require social and political support (e.g. social distancing measures, including teleworking) [2, 3]. On February 25, the Japanese government’s basic policy for COVID-19 control was established; it strongly urges citizens, companies, and local communities to telework and stagger commuting hours [4].

Prime Minister Shinzo Abe declared a state of emergency in 7 of the 47 prefectures on the evening of April 7 [5] and nationwide on April 16. The declaration allows each prefectural government to request residents to refrain from going outside for non-essential reasons, but there is basically no legal means to enforce such regulations. In other words, preventive actions, including social distance measures, are largely dependent on the voluntary efforts of individuals, companies, and organizations.

## Methods

On March 5, 2020, in response to the spread of COVID-19, Kanagawa Prefecture (the second most populous prefecture in Japan after Tokyo, with approximately 9 million people, and one of the prefectures subject to the early declaration of a state of emergency) launched a personalized support program through LINE’s chatbot (Japan’s largest social communication application with 83 million active users, accounting for 65% of the Japanese population) [6]. Geographical scope of the location of Kanagawa Prefecture is presented in Fig. S1. A questionnaire is provided through the application inquiring about the user’s physical condition, and what actions they are currently taking to prevent infections. Based on the content of the initial responses, each user will receive a follow-up questionnaire or be directed to a consultation desk and provided with personalized information on how to prevent the infection. More details of this LINE service can be found elsewhere [7].

We received data from Kanagawa and analyzed the initial response data of 431,106 individuals aged over 15 years until April 16, 2020 to assess the extent to which preventive actions are being taken and how they have changed over a month, including the date of the declaration of a state of emergency in Kanagawa (April 7). Figure S2 shows the cumulative number of respondents by day in the study period.

## Results

**Fig. 1:**
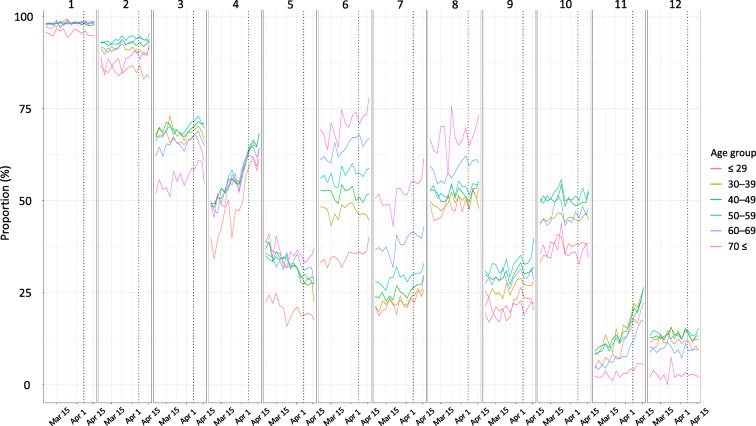
Trends in the implementation rate of preventive actions. Each point is an aggregate value for every 3 days. No adjustment was implemented. 1 Hand washing and gargle; 2 cough etiquette (masks, etc.) 3 hand disinfection with alcohol; 4 regular ventilation; 5 maintaining humidity; 6 a well-balanced diet; 7 regular exercise; 8 getting plenty of rest; 9 avoidance of crowds other than staggered commuting; 10 being feasible to take time off from school or work when having symptoms of a cold, such as a fever; 11 telework; 12 staggered commuting hours. The dotted lines indicate April 7, when the state of emergency was declared

The results show that handwashing and cough etiquette are quite widespread preventive actions, as about 90% of all age groups were practicing them (Fig. 1). On the other hand, only about half of the respondents reported that it was not feasible to take leave in case of cold symptoms, and only about less than 20% of the respondents answered that telework and staggering commuting hours was feasible for them, although a slight improvement was observed during the study period. The declaration of the state of emergency did not have a clear influence on this trend, and excluding those who do not work did not change our conclusions.

**Fig. 2:**
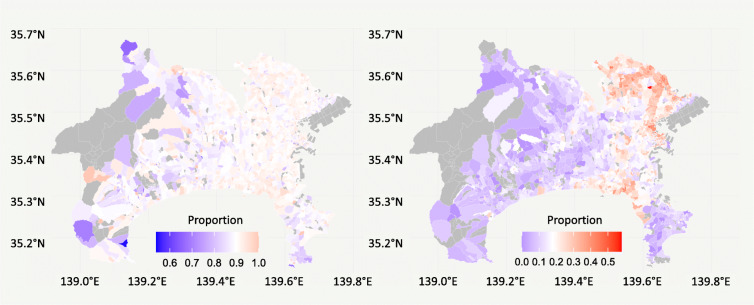
Geographical distribution of the implementation rate of preventive action in the study period. Left panel: proportion of all respondents who performed both hand washing and gargle and cough etiquette (masks, etc.); right panel: proportion of telework implementation among office workers, civil servants, and part-time workers. Both maps are at the post code level, with areas with fewer than 10 respondents excluded from the analysis (gray color). The range of the legends in each panel is that of the regional proportions, with the mid-point (white color) being the average

Figure 2 shows the geographical distribution of the implementation rate of preventive actions. While there was relatively little geographical variation in the implementation of handwashing and cough etiquette, there was a remarkable difference in the implementation of telework between eastern area around the prefectural capital and other areas.

## Discussion

It should be noted that this data was collected from LINE users in Kanagawa who voluntarily responded in the early stages of the program and may not be representative of the population of Japan as a whole, or of Kanagawa Prefecture. It is also necessary to note that this analysis did adjust for confunding variables that may change over time or geographically (e.g., changes in temperature; during the study period, it was spring in Japan and the temperature was on an upward trend). These findings might indicate that for preventive actions that can be implemented with *individual* efforts, many of them have already been successfully implemented. However, preventive actions such as teleworking that require more *social and political* efforts are much more difficult to implement on an individual basis. Therefore, social and political support to promote flexible working practices is an urgent issue that is expected to play a crucial role in curbing the spread of COVID-19 in Japan.

## Electronic Supplementary Material

ESM 1 Geographical scope of the location of Kanagawa Prefecture. (PDF 965 kb)

ESM 2 Daily cumulative number of respondents considered in the analysis. It should be noted that the nationwide LINE surveys led by the Japanese Ministry of Health, Labor and Welfare (MHLW) were conducted on March 31–April 1, April 5–6, April 12–13, and May 1–2 (which are different from the prefecture’s LINE service we used in this study) [8]. For the 2nd and 3rd survey (on April 5–6 and April 12–13), when users responded to the nationwide survey, they also received a LINE message from MHLW encouraging them to respond to the prefecture‘s LINE questionnaire. This should be one of the reasons for the rapid increase in the number of respondents on April 5 and 12. (PDF 23 kb)
