# Supplementary figures and images for: Time to Reconsider Diverse Ways of Working in Japan to Promote Social Distancing Measures against the COVID-19
Source: J Urban Health. 2020 Jun 30;97(4):457–60. doi: 10.1007/s11524-020-00464-4 (PMC7325638; doi:10.1007/s11524-020-00464-4)

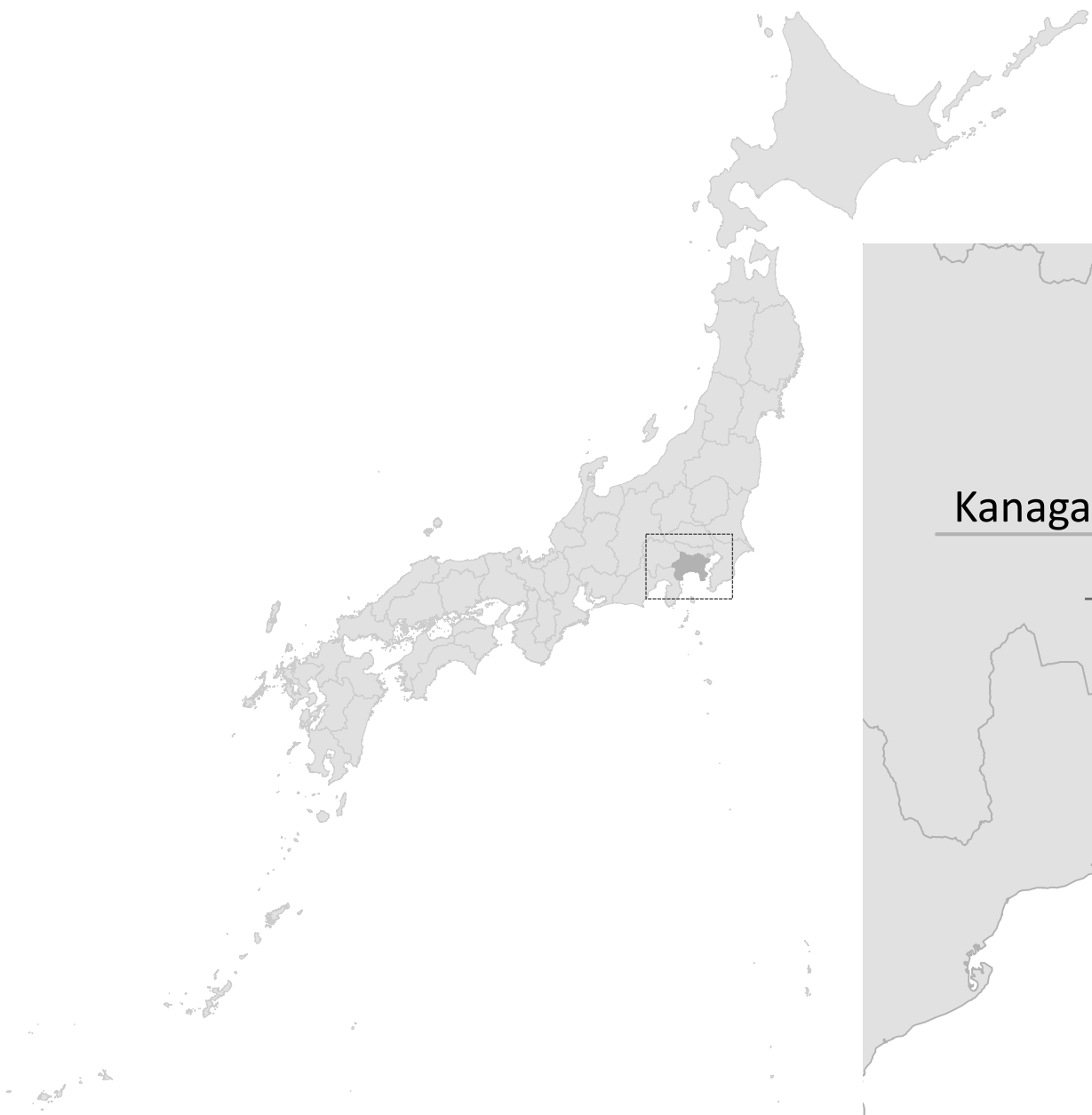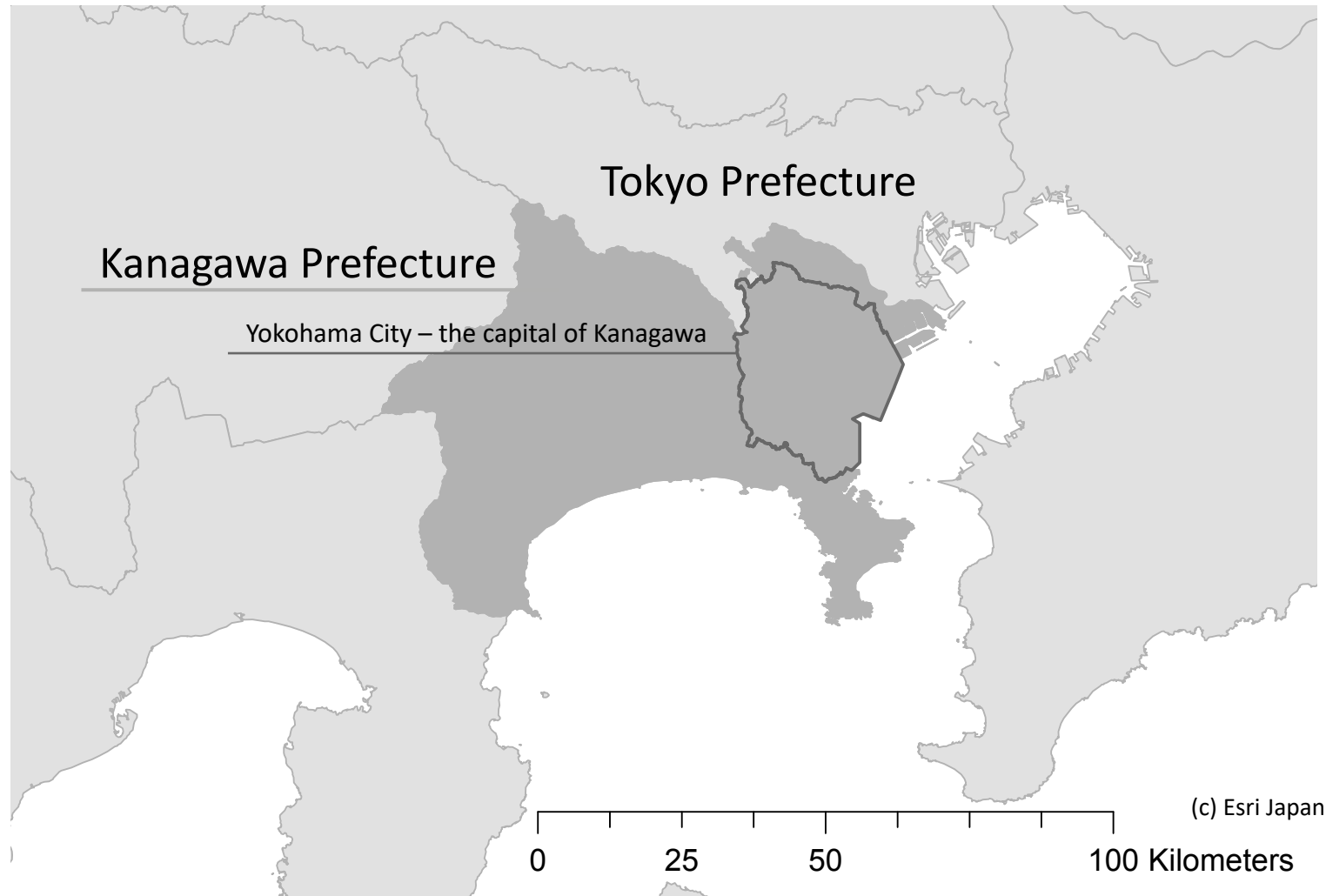

Supplement: Supplementary file 1 — Geographical scope of the location of Kanagawa Prefecture. (PDF 965 kb) [file 11524_2020_464_MOESM1_ESM.pdf]

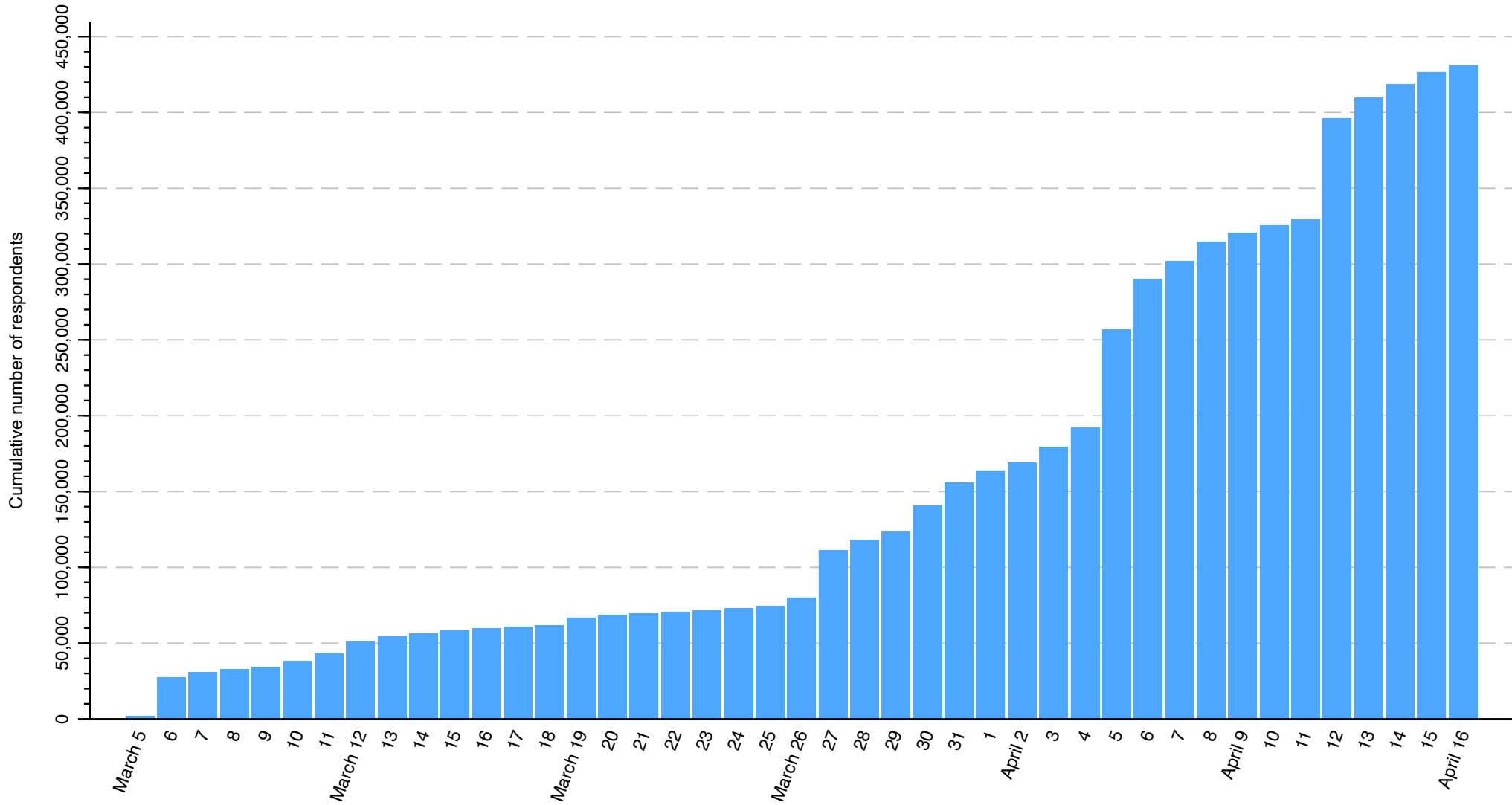

Supplement: Supplementary file 2 — Daily cumulative number of respondents considered in the analysis. It should be noted that the nationwide LINE surveys led by the Japanese Ministry of Health, Labor and Welfare (MHLW) were conducted on March 31–April 1, April 5–6, April 12–13, and May 1–2 (which are different from the prefecture’s LINE service we used in this study) [8]. For the 2nd and 3rd survey (on April 5–6 and April 12–13), when users responded to the nationwide survey, they also received a LINE message from MHLW encouraging them to respond to the prefecture‘s LINE questionnaire. This should be one of the reasons for the rapid increase in the number of respondents on April 5 and 12. (PDF 23 kb) [file 11524_2020_464_MOESM2_ESM.pdf]
